# Evaluation of AJCC staging system and proposal of a novel stage grouping system in retroperitoneal liposarcoma: the Fudan Zhongshan experience

**DOI:** 10.3389/fonc.2024.1373762

**Published:** 2024-03-27

**Authors:** Peidang Fan, Ping Tao, Zhenyu Wang, Jiongyuan Wang, Yingyong Hou, Weiqi Lu, Lijie Ma, Yong Zhang, Hanxing Tong

**Affiliations:** ^1^ Department of General Surgery, Zhongshan Hospital, Fudan University, Shanghai, China; ^2^ First Affiliated Hospital, Anhui University of Science and Technology, Huainan, China; ^3^ Department of Laboratory Medicine, Shanghai Traditional Chinese Medicine-Integrated Hospital, Shanghai University of Traditional Chinese Medicine, Shanghai, China; ^4^ Department of General Surgery, Jinshan Hospital, Fudan University, Shanghai, China; ^5^ Department of Pathology, Zhongshan Hospital, Fudan University, Shanghai, China; ^6^ Department of Liver Surgery, Renji Hospital, School of Medicine, Shanghai Jiaotong University, Shanghai, China; ^7^ Department of General Surgery, Zhongshan Hospital (Xiamen), Fudan University, Xiamen, China; ^8^ Xiamen Clinical Research Center for Cancer Therapy, Xiamen, China

**Keywords:** revised T stage, modified TNM system, os, RPLS, FNCLCC

## Abstract

**Background:**

Overall survival (OS) varies significantly among individuals with heterogeneous retroperitoneal liposarcoma (RPLS), even among those with the same clinical stage. Improved staging of RPLS is a critical unmet need, given the disappointing results of external validations of the 8^th^ American Joint Committee on Cancer (AJCC) TNM staging system.

**Methods:**

The cohort study included 220 consecutive patients who underwent surgical resection for primary RPLS at the largest sarcoma centre of Fudan University in China from September 2009 to August 2021, combined with 277 adult patients with RPLS in the SEER database from 1975 to 2020. Data analysis was performed from December 2021 to December 2022. Patients were retrospectively restaged according to the 8th and 7th editions of the TNM staging system as well as the new TNM (nTNM) staging system. The primary endpoint was overall survival (OS). Comparative analysis of postoperative survival was performed using the Kaplan-Meier method, and differences between subgroups were tested using the log-rank test. The OS prediction nomogram was generated based on baseline variables and tumour characteristics. Harrell’s consistency index (C-index), area under the curve (AUC) of receiver operating characteristic curves (ROC), and calibration curves were used to evaluate the performance of the nomogram.

**Results:**

A total of 497 patients were enrolled in the study, including 282 (56.7%) male patients. The median follow-up was 51 months (interquartile range, IQR, 23-83), and the OS rates at 1, 3, and 5 years were 87.9%, 75.3%, and 64.9%, respectively. According to the staging distribution of the AJCC 7th edition, 6 patients were stage IA (1.2%), 189 patients were stage IB (38%), 12 patients were stage IIA (2.4%), 150 patients were stage IIB (30.1%), 131 patients were stage III (26.3%), and 9 patients were stage IV (1.8%). With the 8th edition staging, this distribution changed: 6 patients (1.2%) were stage IA, 189 patients (38%) were stage IB, 12 patients (2.4%) were stage II, 24 patients (4.8%) were stage IIIA, 257 patients (51.7%) were stage IIIB, and 9 patients (1.8%) were stage IV. 182 patients (36.6%) were reclassified according to the nTNM staging system with the new T stage classification. The C-index and log-rank score improved after implementation of nTNM implementation. The nTNM system was associated with improved identification of high-risk patients compared with the AJCC 7^th^ and 8^th^ TNM. The FNCLCC stage proved to be highly prognostic with significant intergroup differences in OS. The calibration curve shows a high degree of agreement between the actual OS rate and the nomogram estimated OS rate.

**Conclusion:**

Compared with 8^th^ AJCC TNM, 7^th^ AJCC TNM staging system showed a more homogeneous staging distribution and a slight improvement in the prognostic accuracy of RPLS. The revised T-stage and nTNM systems showed better risk stratification performance. The FNCLCC stage was found to have high prognostic value, further emphasising histological grade is the least negligible prognostic factor in predicting patient survival. The constructed nomogram model enables individualized prognostic analysis and helps to develop risk-adapted therapy for RPLS patients.

## Introduction

The relative rarity and biological heterogeneity of soft tissue sarcoma (STS), especially in retroperitoneal sarcoma (RPS) (1), contributes to the potential diversity and complexity of the disease, thereby limiting the development of robust histiotype-specific or site-specific evidence to guide clinical management. However, the increasing recognition of RPS over the past few decades has led to standard classification, grading and staging systems (2).

The most commonly used grading system is the (Fédération Nationale des Centres de Lutte Contre le Cancer) FNCLCC grading system for STS, and the most commonly used nomograms are derived from Sarculator, Memorial Sloan-Kettering Cancer Center (MSKCC) (3), and Gronchi A (4). However, all of these nomograms are not specific for retroperitoneal liposarcoma (RPLS), as only 13% of patients in the development and validation cohorts of the MSKCC database were RPLS patients (5, 6), whereas primary extremity sarcomas in Sarculator (7) and RPS in Gronchi A (4). Notably, STS with different pathologic types and anatomic sites exhibit an extremely high degree of tumour heterogeneity and biological behavior (8). Furthermore, most of these models have not been included in the TNM staging system, and the external validation power of these models is limited (9, 10). Therefore, histology-type-specific clinical staging and nomograms are needed to stratify patients with STS, especially for RPLS.

At the same time, the latest 8th edition of the AJCC STS staging system recognizes the importance of the anatomic location of the sarcoma and establishes a site-specific staging system that distinguishes RPS from other sarcomas (11), based primarily on tumour size without any clinical experience or published evidence, but completely ignoring the important predictive information tumour invasion of adjacent organs (12), tumour multifocality (13), and histologic subtype (14). A recent evaluation of the performance of the 8th edition of the AJCC staging system for RPS using a large national database showed that its overall prognostic performance remains unsatisfactory (12). Therefore, the ability of the new staging system to risk stratify specific types (e.g., liposarcoma) among RPS needs to be investigated.

The aim of this study was to compare the clinical presentation of AJCC 7th TNM staging system and AJCC 8th TNM staging system in RPLS using data extracted from the SEER database in conjunction with the Fudan University database. Meanwhile, we aimed to propose a revised T staging algorithm based on our clinical experience and also to construct a novel nomogram incorporating several indispensable clinical factors for personalized risk assessment in RPLS.

## Methods

### Retrospective patient cohort

Patients undergoing radical resection for RPLS were identified retrospectively from the SEER and Fudan databases. This is a population-based study of subjects from a publicly available de-identified patient database that does not require institutional review board approval. This study was reviewed and approved by the Institutional Review Board (ID: B2022-586R) of Zhongshan Hospital, Shanghai, China. All studies were guided by the principles of the Declaration of Helsinki.

Variables downloaded from the SEER database (www.seer.cancer.gov) included age, sex, year of diagnosis, tumour site, tumour diameter, lymph node metastasis, distant metastasis, histological subtype of sarcoma, histological grade, survival information, and follow-up data.

For the sarcoma centre at the ZSFD, a dynamically updated big data database has been developed includes daily electronic medical record data. Sarcoma patients treated at the ZSFD since September 2009 were included in the database. Each patient’s data were collected from 10 electronic health record systems, including the outpatient work system, pathology system, electronic medical record, the follow-up system, laboratory information system, electrocardiogram system, anaesthesia information management system, hospital information system, physical examination information system, tumour tissue biobank.

Inclusion criteria included the following: (1) RPLS on pathologic examination; (2) complete TNM staging information (**Supplementary Table 1**); (3) complete clinical information; (4) no history of other malignant tumours; (5) effective postoperative follow-up information.

### Clinical end point and follow-up

The endpoint of the current study was OS, defined as the time from surgery to death from any cause (15). OS was prospectively collected from the sarcoma centre database. Patients were followed up over time through medical records and telephone calls. Follow-up time was defined as the interval between the date of surgery and the time of the last follow-up or death.

Follow-up consisted of a physical examination, laboratory tests, and at least one radiological imaging test [abdominal computed tomography (CT) and/or magnetic resonance imaging (MRI)] every 3 months for the first 2 years after surgery, then every 6 months, and once a year after 5 years, depending on the specific pathological subtype. PET-CT was not routinely performed. Patients were also contacted by telephone if necessary.

### nTNM staging system

Patients were restaged based on pathological tumour size, nodal status and metastatic spread to distant sites according to the 7th (16) and 8th edition staging systems (11). The nTNM system incorporated a modified T-stage (21cm, the median value from tumour diameter in the ZSFD database) into the 7th and 8th editions, respectively, and patients were also restaged according to sub-stage re-grouping based on prognostic performance on surveillance.

### Statistical analysis

Continuous variables were expressed as mean with standard deviation (SD). Categorical variables were expressed as frequencies and percentages. Fisher’s exact test and chi-squared test were used for categorical data. All tests were 2-tailed.

Kaplan-Meier curves were plotted to estimate median OS. Cox proportional hazards regression models were applied to identify OS-related prognostic factors The assumptions of the Cox model were tested by partial residual analysis.

The accuracy of the 7^th^ TNM and 8^th^ TNM staging systems in predicting postoperative OS was compared using concordance index, ROC and AUC.

The nomogram predicting postoperative OS was generated from the results of Cox regression analysis. The corresponding calibration curves were used to compare the predicted probabilities of the nomograms with the agreement between the observations.

This study was conducted using statistical software including SPSS (version 26.0), R software (version 4.0.3), and GraphPad Prism (version 8.0), and a two-sided p-value of less than 0.05 was considered statistically significant. All methods followed relevant guidelines and regulations.

## Results

### Baseline characteristics

A total of 497 consecutive RPLS were retrospectively included in the follow-up analysis, with 277 patients from the SEER database and 220 patients from the ZSFD database (**Supplementary Figure 1**). Baseline and tumour characteristics are demonstrated in **Table 1**. 282 (56.7%) were male and the median age at diagnosis was 48 years (range, 19-85 years). The median tumour size was 21 cm (IQR, 15-30 cm). Of note, only 1% of patients had lymph node metastases in the overall cohort and 1.8% had distant metastases.

**Table 1:** Baseline characteristics of SEER and ZSFD cohorts.

Characteristics	No. (%)	SEER Cohort	ZSFD Cohort
	Total Cohort		
	(N=497)	(N=277)	(N=220)
**Age, median (IQR)**	61 (52-68)	64 (55-70)	56 (50-65)
Sex			
Male	282 (56.7%)	156 (56.3%)	126 (57.3%)
Female	215 (43.3%)	121 (43.7%)	94 (42.7%)
**Tumor size, median (IQR), mm**	210 (150-300)	200 (130-270)	245 (170-300)
AJCC 7th T Stage			
T1	19 (3.8%)	15 (5.4%)	4 (1.8%)
T2	478 (96.2%)	262 (94.6%)	216 (98.2%)
AJCC 8th T Stage			
T1	19 (3.8%)	15 (5.4%)	4 (1.8%)
T2	38 (7.6%)	28 (10.1%)	10 (4.6%)
T3	79 (16%)	46 (16.6%)	33 (15%)
T4	361 (72.6%)	188 (67.9%)	173 (78.6%)
N Stage			
N0	492 (99%)	272 (98.2%)	220 (100%)
N1	5 (1%)	5 (1.8%)	0
M Stage			
M0	488 (98.2%)	270 (97.5%)	218 (99.1%)
M1	9 (1.8%)	7 (2.5%)	2 (0.9%)
FNCLCC Grade			
1	201 (40.4%)	104 (37.5%)	97 (44.1%)
2	159 (32%)	75 (27.1%)	84 (38.2%)
3	137 (27.6%)	98 (35.4%)	39 (17.7%)
Histologic subtypes			
WDLPS	192 (38.6%)	82 (29.6%)	110 (50%)
DDLPS	221 (44.5%)	154 (55.6%)	67 (30.5%)
MLPS	23 (4.6%)	10 (3.6%)	13 (5.9%)
PLS	9 (1.8%)	6 (2.2%)	3 (1.4%)
Mixed liposarcoma	21 (4.2%)	2 (0.7%)	19 (8.6%)
Liposarcoma, NOS	31 (6.2%)	23 (8.3%)	8 (3.6%)

WDLPS, well-differentiated liposarcoma; DDLPS, dedifferentiated liposarcoma; MLPS, myxoid Liposarcoma, PLS, pleomorphic liposarcoma; NOS, Not specified.

Due to the time span of the study, 7th or even 6th edition of AJCC TNM staging was applied before 2017 and 8th edition after 2017, in order to effectively evaluate the usability of 7th and 8th edition, therefore all patients were restaged to obtain their respective 7th and 8th edition staging. As shown in **Table 2**, when the 7th edition of the TNM staging system was applied, the numbers of patients with stage IA, IB, IIA, IIB, III, and IV were 6 (1.2%), 189 (38%), 12 (2.4%), 150 (30.1%) 131 (26.3%) and 9 (1.8%), respectively. This distribution changed after the application of the 8th edition of the classification: 6 patients (1.2%) were in stage IA, 189 patients (38%) were in stage IB, 12 patients (2.4%) were in stage II, 24 patients (4.8%) were in stage IIIA, 257 patients (51.7%) were in stage IIIB, and 9 patients (1.8%) were in stage IV. Using the 8th edition classification, 293 patients (58.9%) were reclassified to another stage.

**Table 2:** Staging reclassification by the 7th and 8th Edition of the TNM and nTNM staging system for RPLS.

Overall Stage	Patients, No. (%)	TNM 8th Edition	nTNM 7th Edition	nTNM 8th Edition
	TNM 7th Edition			
Stage I				
IA	6 (1.2)	6 (1.2)	94 (18.9)	48 (9.6)
IB	189 (38)	189 (38)	101 (20.3)	147 (29.6)
Stage II				84 (16.9)
IIA	12 (2.4)	12 (2.4)	153 (30.8)	
IIB	150 (30.2)	NA	83 (16.7)	NA
Stage III	131 (26.4)	NA	57 (11.5)	NA
IIIA	NA	24 (4.8)	NA	69 (13.9)
IIIB	NA	257 (51.7)	NA	140 (28.2)
Stage IV	9 (1.8)	9 (1.8)	9 (1.8)	9 (1.8)

NA, not applicable; TNM, tumor node metastasis; RPLS, retroperitoneal liposarcoma.

Due to the time span of the study, 7th or even 6th edition of AJCC TNM staging was used before 2017 and 8th edition after 2017, in order to effectively evaluate the performance of 7th and 8th edition, therefore all patients were restaged to obtain their respective 7th and 8th edition staging.

Regarding other pathological variables, a total of 201 patients had a G1 tumour (40.4%), 159 patients had a G2 tumour (31.9%) and 137 patients had a G3 tumour (27.5%). The proportion of each histological subtype was 38.6% (WDLPS), 44.5% (DDLPS) and other LPS (16.9%).

### Clinical outcomes by TNM staging system

**Figure 1:**
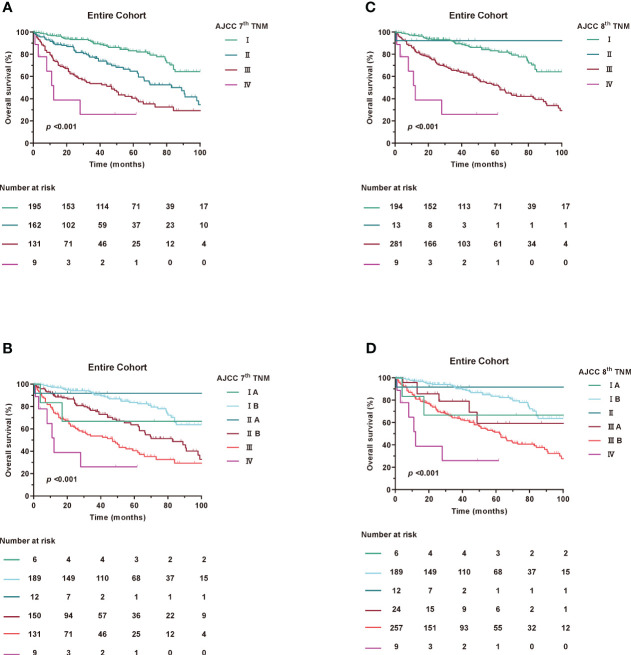
Kaplan-Meier curves of OS in Entire Cohort according to AJCC TNM staging. **(A, B)** AJCC 7th TNM staging, **(C, D)** AJCC 8th TNM staging.

At the last follow-up, 336 patients (67.6%) were alive and the median follow-up for the entire cohort was 51 (IQR, 23-83) months. The median OS for the entire cohort was 85 (95%CI, 73-97) months, with the 1-, 3-, and 5-year OS estimates of 87.9%, 75.3%, and 64.9%, respectively. Kaplan-Meier curves for OS were examined according to the 7th and 8th editions of the AJCC TNM (**Figure 1**).

Based on Kaplan-Meier survival analysis, the 5-year survival rates changed from 83.2% for stage I, 64.7% for stage II, 40.5% for stage III and <25.9% for stage IV (log-rank, p<0.001) under the 7^th^ AJCC TNM to 83.1% for stage I, NA for stage II, 52.2% for stage III and <25.9% for stage IV (log-rank, p<0.001) under the 8^th^ AJCC TNM. Similar results were found in the SEER and ZSFD cohorts (**Supplementary Figure 2**).

In the subgroup of patients, neither 7th or 8th edition T stage was discriminative for survival (**Supplementary Figure 3**). Furthermore, no significant changes in N and M stage were observed between the 7th and 8th editions (data not shown).

When assessing the prognostic accuracy for OS, the C-index reached 0.694 (95% CI, 0.673-0.715) for 7^th^ AJCC TNM and 0.654 (95% CI, 0.635-0.672) for 8^th^ AJCC TNM staging system (**Table 3**). The AUC value at 1, 3 and 5 years were 74.5%, 72% and 70.7% for the 7th edition and 70.2%, 68.5% and 67.7% for the 8th edition for OS as shown in **Supplementary Figure 4**. The time-dependent AUCs indicated that 7th has higher AUCs compared to 8th of the OS. Consistent with the overall cohort, similar results were found in the SEER and ZSFD cohorts, respectively (**Supplementary Figure 5**).

**Table 3:** Summary of the C-index of prognostic models for OS in patients with RPLS.

Models	C-index (95% CI)	*p* value
**7th TNM Stage**	0.696 (0.675-0.717)	< 0.001
**8th TNM Stage**	0.664 (0.645-0.683)	< 0.001
**7th nTNM Stage**	0.677 (0.656-0.699)	< 0.001
**8th nTNM Stage**	0.676 (0.655-0.696)	< 0.001
**FNCLCC Grade**	0.683 (0.662-0.704)	< 0.001

TNM, tumor node metastasis; FNCLCC, French Fédération Nationale des Centres de Lutte contre le Cancer.

Taken together, these results demonstrate that the 7th edition has a more even staging distribution and a slightly improved prognostic accuracy for RPLS compared to the 8th edition.

### Proposed modifications to the 7th and 8th edition

**Figure 2:**
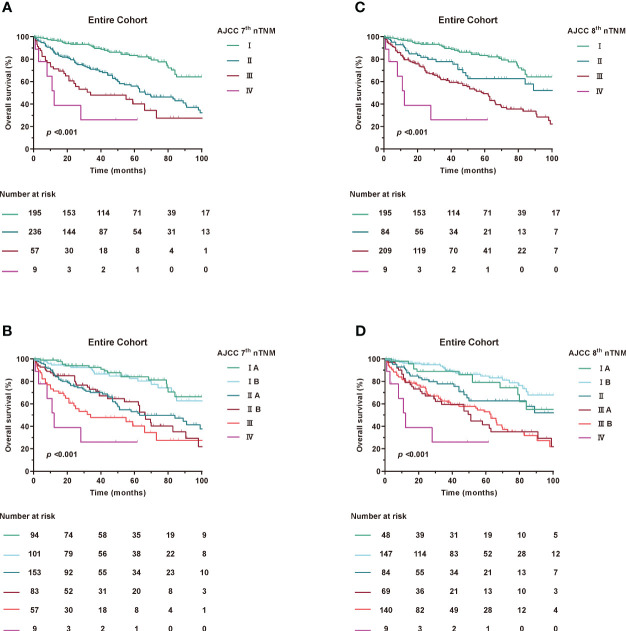
Kaplan-Meier curves of OS in Entire Cohort according to modified AJCC TNM staging. **(A, B)** AJCC 7th nTNM staging, **(C, D)** AJCC 8th nTNM staging.

Accumulating evidence has shown that RPLS, unlike other solid tumours, is located in the retroperitoneal space without any obvious clinical features during tumour progression (17, 18). In addition, patients were always diagnosed with a large tumour volume and an overloaded tumour burden (2). We then estimated the value of T stage in prognostic accuracy for OS. Interestingly, patients with tumour diameters of less than 5 cm were extremely rare, both in the 7th edition of stage IA and IIA and the 8th edition of stage IA and II (**Figure 2**), which had greatly attracted our attention.

Patients were restaged according to the newly proposed tumour size staging, which is based on median and quartile of the entire cohort. For the 8th edition staging criteria, T1: ≤15 cm maximum diameter, T2: >15 to 21 cm maximum diameter, T3: >21 cm to 30 cm maximum diameter, and T4: >30 cm. Meanwhile, for the 7th edition staging criteria, T1: ≤21 cm maximum diameter, T2: >21 cm maximum diameter.

Using the new modified T-stage classification and regrouping the TNM staging, the distribution of patients was optimised for stage IA in 48 patients (9.6%), stage IB in 147 (29.5%), stage II in 84 (16.9%), stage IIIA in 69 (13.8%), stage IIIB in 140 (28.1%) and stage IV in 9 (1.8%) in the 8th edition. Meanwhile, according to the AJCC 7^th^ TNM, stage IA was found in 94 patients (18.9%), stage IB in 101 (20.3%), stage IIA in 153 (30.7%), stage IIB in 83 (16.7%), stage III in 57 (11.4%), and stage IV in 9 (1.8%) (**Supplementary Table 2**).

When assessing the prognostic accuracy of the new T stage on OS, the C-index was 0.675 (95% CI, 0.655-0.696) for the modified 7th edition and 0.673 (95% CI, 0.653-0.693) for the modified 8th edition (**Table 3**). The ROC curve for predicting OS at 1, 3 and 5 years showed an AUC of 73.3%, 70.7% and 69% for the modified 7th edition and an AUC of 72.6%, 69.5% and 68.4% for the modified 8th edition, as shown in **Supplementary Figure 6**.

To further explore the prognostic predictive power of the nTNM staging in different histological subtypes of RPLS, we performed further analyses. Since the main component was on WDLPS and DDLPS, we empirically divided the WDLPS, mixed liposarcoma and ‘liposarcoma, NOS’ into a group named H1, and the remaining into a group named H2. The results of the study showed that the C-indexes of H1 in 7th nTNM and 8th nTNM staging 0.632 and 0.643, whereas the C-indexes of H2 were 0.607 and 0.590. The ROC curves for the 7th nTNM and 8th nTNM staging in H1 and H2 predicting 1-, 3-, and 5-year postoperative survival are shown in the **Supplementary Figure 7**.

Taken together, these results suggest that the overall model fit of the nTNM staging is better compared with 7th TNM and 8th TNM staging and that the new T staging can be used as a powerful tool for RPLS risk stratification.

### FNCLCC grade in prognosis

**Figure 3:**
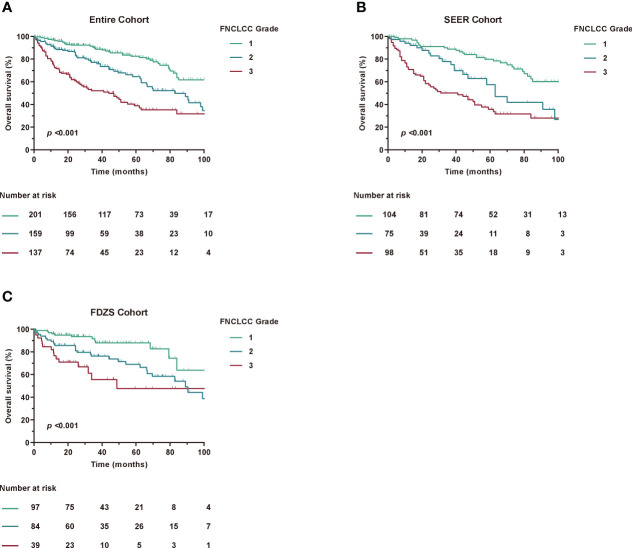
Kaplan-Meier curves of OS according to FNCLCC Grade among different cohorts. **(A)** Entire Cohort, **(B)** SEER Cohort, **(C)** FDZS Cohort.

Since histologic grading has been shown to be one of the factors most strongly associated with postoperative prognosis in patients with RPLS (12, 19, 20), we also examined the association between the modified 7th and 8th edition histological grades. Notably, histological grade analysis based on FNCLCC grading remained unchanged. In addition, the C-index for FNCLCC grade alone was 0.683 (95% CI, 0.662-0.704) for prognostic accuracy (**Table 3**). Furthermore, the FNCLCC grade showed a statistically significant survival difference in risk stratification compared with the TNM staging system (**Figure 3**), as confirmed by the results of the SEER and ZSFD cohorts. However, when assessing the prognostic accuracy for OS in the combination of FNCLCC grade and TNM, the C-index reached 0.710 (95% CI, 0.688-0.732).

Taken together, these results showed that the TNM staging system had some advantages over FNCLCC grade alone, but needed to be improved in a larger RPLS cohort.

### Nomogram development and validation

**Figure 4:**
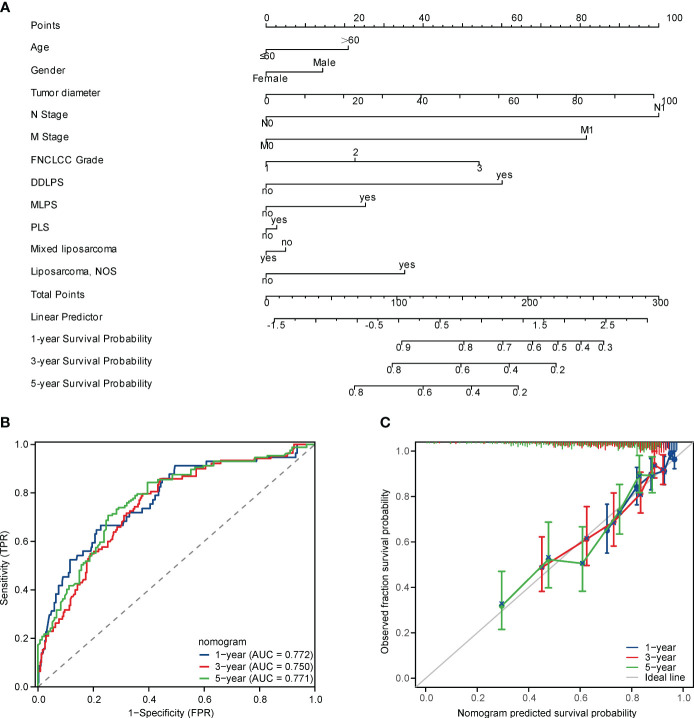
**(A)** A nomogram for predicting the 1-, 3-, 5-year OS in RPLS after resection, **(B)** Receiver operating characteristic (ROC) curves of the nomogram in predicting 1-, 3-, 5-year OS in RPLS after resection, **(C)** Calibration plots of the nomogram in predicting 1-, 3-, 5-year OS in RPLS after resection.

Nomogram is a concise graphical model that integrates multiple prognostic-related factors and is an effective tool for personalized risk assessment of cancer patients (21, 22). To further explore the significant differences in baseline variables and tumour characteristics, we constructed the OS-related nomogram, including sex, age, tumour size, FNCLCC grade pathological subtypes, lymph node metastasis and distant metastasis (**Figure 4A**). Of note, the C-index reached 0.726 (95% CI, 0.705-0.748) and the AUC values at 1, 3, and 5 years postoperatively were 77.2%, 75%, and 77.1%, respectively (**Figure 4B**). The calibration curve showed that the actual OS rate was highly consistent with the nomogram estimated OS rate (**Figure 4C**).

## Discussion

Tumour heterogeneity requires personalised cancer therapy (23–26). However, RPLS is one of the most heterogeneous types of solid tumours (27). Determining a patient’s postoperative risk can assist oncologists in developing the most appropriate personalized treatment plan (28). However, the broad nature of the TNM staging system and the heterogeneity of patients with tumours in the same stage make its widespread use for risk stratification still imperfect.

Furthermore, RPLS was particularly influenced by anatomical location, tumour biological behaviour, histological grade and pathological subtype (12, 29–31). Given its rarity, heterogeneity and heterogeneous therapeutic response, the true predictors of survival and staging system for RPLS are still under investigation.

Relative to the 7^th^ AJCC TNM, the 8^th^ AJCC TNM addresses this issue by providing separate staging algorithms according to the anatomic location of the sarcoma, such as limbs and trunk, retroperitoneum, or head and neck (11). However, no significant improvement was found, which may be related to the behaviour of different histological types of sarcomas (32).

Several studies have now evaluated the 8^th^ AJCC TNM staging system (33, 34). However, categorizing continuous data generates regression coefficients that are weighted according to the distribution of data within each category, which almost always fails to capture the true nonlinear relationship between a continuous variable and its log hazard (35). In any case, risk stratification for lymph node metastasis (LNM) is unlikely to play a major determining role in prognostic model performance, as less than 5% of sarcoma patients develop nodal metastases (32).

To the best of our knowledge, studies that have modified the T-staging in the TNM staging system and developed a comprehensive nomogram for estimating OS are lacking. This study proposes a nomogram that combines the TNM staging system and other widely assessed clinical characteristics to accurately assess the optimal stratification of patients with RPLS.

Compared with the widely used Sarculator (3) and the multicentre nomogram proposed by Gronchi A (4), the nomogram proposed in this study has comparable predictive performance and is unique to RPLS. The C index of the sarculator and the multicentre nomogram proposed by Gronchi A in RPS is 0.73 (6) and 0.68 (4), respectively. The nomogram proposed in this study had excellent discriminatory power (C index 0.726; 95% CI 0.705-0.748) and the actual OS was highly consistent with the probability of OS estimated by the nomogram, which was confirmed by the calibration curves, which represents a reliable model with strong predictive performance for OS estimation in RPLS.

A number of nomograms for predicting survival in STS or RPLS have been available in the published literature since 2002 (36–38), but unfortunately most of them were not covered by the TNM staging system. To fill this gap, we conducted a large real-world study including 220 primary RPLS patients from the ZSFD database and 277 RPLS from the SEER database to develop and validate a nomogram for estimating OS.

## Limitations

There exist some limitations of the research that cannot be ignored. Firstly, selection bias may be unavoidable due to the retrospective cohort study design. Second, although the internal validation cohort of the SEER database showed excellent discriminatory power with a high degree of concordance between the actual OS and the estimated OS probability of the nomogram, which was confirmed by the calibration curves, we did not perform external validation in China or the United States (Unite State of America). Third, this study was conducted in a high-flow sarcoma centre and may not be generalizable to a small population of RPLS patients.

## Conclusions

Compared with 8^th^ AJCC TNM, 7^th^ AJCC TNM staging system showed a more homogeneous staging distribution and a slight improvement in the prognostic accuracy of RPLS. The revised T-stage and nTNM systems showed better risk stratification performance. The FNCLCC stage was found to have high prognostic value, further emphasising histological grade is the least negligible prognostic factor in predicting patient survival. The constructed nomogram model enables individualized prognostic analysis and helps to develop risk-adapted therapy for RPLS patients.

## Data availability statement

The data that support the findings of this study are available on request from corresponding author.

## Ethics statement

The studies involving humans were approved by Institutional Review Board of Zhongshan Hospital, Shanghai, China (ID: B2022-586R). The studies were conducted in accordance with the local legislation and institutional requirements. Written informed consent for participation was not required from the participants or the participants’ legal guardians/next of kin in accordance with the national legislation and institutional requirements.

## Author contributions

PF: Conceptualization, Data curation, Formal analysis, Methodology, Validation, Writing – original draft, Writing – review & editing. PT: Conceptualization, Formal analysis, Methodology, Validation, Writing – original draft. ZW: Conceptualization, Data curation, Formal analysis, Investigation, Methodology, Writing – original draft. JW: Conceptualization, Data curation, Supervision, Writing – original draft. YH: Supervision, Writing – review & editing. WL: Resources, Supervision, Writing – review & editing. LM: Conceptualization, Formal analysis, Methodology, Resources, Supervision, Writing – original draft, Writing – review & editing. YZ: Conceptualization, Methodology, Resources, Supervision, Writing – review & editing. HT: Conceptualization, Formal analysis, Methodology, Resources, Supervision, Validation, Writing – original draft, Writing – review & editing.
