# Supplementary material for: Evaluation of AJCC staging system and proposal of a novel stage grouping system in retroperitoneal liposarcoma: the Fudan Zhongshan experience
Source: Front Oncol. 2024 Mar 27;14:1373762. doi: 10.3389/fonc.2024.1373762 (PMC11004455; doi:10.3389/fonc.2024.1373762)
Supplement: Supplementary file 1 [file DataSheet_1.docx]

**Supplementary Online Content**

**Title: Evaluation of AJCC staging system and proposal of a novel stage grouping system in retroperitoneal liposarcoma: The Fudan Zhongshan Experience**

**Authors**: Peidang Fan^1†^ MD, Ping Tao^2†^ MD, Zhenyu Wang^3†^ MD, Jiongyuan Wang^4†^  MD, Yingyong Hou^5^ MD, PhD, Weiqi Lu^4^ MD, Lijie Ma^6^* MD, PhD, Yong Zhang^4^* MD, PhD, Hanxing Tong^1,4^* MD, PhD

**Supplementary Table 1.** Definition of RPLS in the 7th and 8th Editions of the American Joint Committee with the TNM staging system.

**Supplementary Table 2.** Cross-tabulation of the 7th and 8th Edition with the nTNM stage system.

**Supplementary Figure 1.** Flowchart of patient selection in two Cohorts. (A) SEER Cohort, (B) FDZS Cohort.

**Supplementary Figure 2.** Kaplan-Meier curves of OS according to AJCC TNM staging in two Cohorts. (A,B) AJCC 7^th^ TNM staging for SEER Cohort, (C,D) AJCC 8^th^ TNM staging for SEER Cohort, (E,F) AJCC 7^th^ TNM staging for FDZS Cohort, (G,H) AJCC 8^th^ TNM staging for FDZS Cohort.

**Supplementary Figure 3.** Kaplan-Meier curves of OS by AJCC 7^th^ T staging. (A,B,C) and AJCC 8^th^ T staging (D,E,F) among different Cohorts. (A,D) Entire Cohort, (B,E) SEER Cohort, (C,F) FDZS Cohort.

**Supplementary Figure 4.** Receiver operating characteristic (ROC) curves in predicting 1-, 3-, 5-year OS in RPLS after resection by AJCC TNM staging. (A) AJCC 7^th^ TNM staging, (B) AJCC 8^th^ TNM staging.

**Supplementary Figure 5.** Time-dependent AUC for the 7th and 8th Edition of TNM Staging system among three Cohorts. (A) Entire Cohort, (B) SEER Cohort, (C) FDZS Cohort.

**Supplementary Figure 6.** Receiver operating characteristic (ROC) curves in predicting 1-, 3-, 5-year OS in RPLS after resection by modified AJCC TNM staging. (A) AJCC 7^th^ nTNM staging, (B) AJCC 8^th^ nTNM staging.

**Supplementary Figure 7.**Receiver operating characteristic (ROC) curves in predicting 1-, 3-, 5-year OS in H1 (WDLPD, Mixed liposarcoma and ‘liposarcoma,NOS’) and H2 (DDLPS, MLPS and PLS) after resection by modified AJCC TNM staging. (A) AJCC 7^th^ nTNM staging for H1, (B) AJCC 8^th^ nTNM staging for H1, (C) AJCC 7^th^ nTNM staging for H2, (D) AJCC 8^th^ nTNM staging for H2.

**Supplementary Table 1.** Definition of RPLS in the 7th and 8th Editions of the American Joint Committee with the TNM staging system.

| Stage | Description |
| --- | --- |
| 7th edition, stages in the T, N, M and G stages | |
| T1a | Maximum tumor diameter ≤ 5 cm, superficial |
| T1b | Maximum tumor diameter ≤ 5 cm, deep |
| T2a | Maximum tumor diameter > 5 cm, superficial |
| T2b | Maximum tumor diameter > 5 cm, deep |
| N0 | No regional lymph node metastasis |
| N1 | Regional lymph node metastasis |
| M0 | No distant metastasis |
| M1 | Distant metastasis |
| 8th edition, stages in the T, N, M and G stages | |
| T1 | Maximum tumor diameter ≤ 5 cm |
| T2 | Maximum tumor diameter > 5 cm and ≤ 10 cm |
| T3 | Maximum tumor diameter > 10 cm and ≤ 15 cm |
| T4 | Maximum tumor diameter > 15 cm |
| N0 | No regional lymph node metastasis or unknown lymph node status |
| N1 | Regional lymph node metastasis |
| M0 | No distant metastasis |
| M1 | Distant metastasis |
| 7th edition staging groups^a^ | |
| Stage ⅠA | T1a/b; N0; M0; G1 |
| Stage ⅠB | T2a/b; N0; M0; G1 |
| Stage ⅡA | T1a/b; N0; M0; G2/3 |
| Stage ⅡB | T2a/b; N0; M0; G2 |
| Stage Ⅲ | T2a/b; N0; M0; G3 |
|  | Any T; N1; M0; Any G |
| Stage Ⅳ | Any T; Any N; M1; Any G |
| 8th edition staging groups^b^ | |
| Stage ⅠA | T1; N0; M0; G1 |
| Stage ⅠB | T2, T3, T4; N0; M0; G1 |
| Stage Ⅱ | T1; N0; M0; G2/3 |
| Stage Ⅲ A | T2; N0; M0; G2/3 |
| Stage Ⅲ B | T3, T4; N0; M0; G2/3 |
|  | Any T; N1; M0; Any G |
| Stage Ⅳ | Any T; Any N; M1; Any G |

^a^Used with the permission of the American Joint Committee on Cancer (AJCC), Chicago, IL. The original source for this material is the AJCC Cancer Staging Manual, Seventh Edition (2010) published by Springer Science and Business Media LLC, www.springer.com.

^b^Used with the permission of the American Joint Committee on Cancer (AJCC), Chicago, IL. The original source for this material is the AJCC Cancer Staging Manual, Eighth Edition (2017) published by Springer Science and Business Media LLC, www.springer.com.

**Supplementary Table 2.** Cross-tabulation of the 7th and 8th Edition with the nTNM stage system.

| nTNM Stage 7th Edition | | nTNM Stage 8th Edition, No. (%) | | | | | | |  |  |  |  |  |  |
| --- | --- | --- | --- | --- | --- | --- | --- | --- | --- | --- | --- | --- | --- | --- |
|  |  | ⅠA |  | ⅠB |  | Ⅱ |  | Ⅲ A |  | Ⅲ B |  | Ⅳ |  | Total |
| ⅠA |  | 48 (9.6) |  | 46 (9.3) |  | 0 |  | 0 |  | 0 |  | 0 |  | 94 (18.9) |
| ⅠB |  | 0 |  | 101 (20.3) | | 0 |  | 0 |  | 0 |  | 0 |  | 101 (20.3) |
| ⅡA |  | 0 |  | 0 |  | 84 (16.9) |  | 69 (13.9) |  | 0 |  | 0 |  | 153 (30.8) |
| ⅡB |  | 0 |  | 0 |  | 0 |  | 0 |  | 83 (16.7) |  | 0 |  | 83 (16.7) |
| Ⅲ |  | 0 |  | 0 |  | 0 |  | 0 |  | 57 (11.5) |  | 0 |  | 57 (11.5) |
| Ⅳ |  | 0 |  | 0 |  | 0 |  | 0 |  | 0 |  | 9 (1.8) |  | 9 (1.8) |
| Total |  | 48 (9.6) | | 147 (29.6) | | 84 (16.9) |  | 69 (13.9) |  | 140 (28.2) |  | 9 (1.8) |  | 497 (100) |


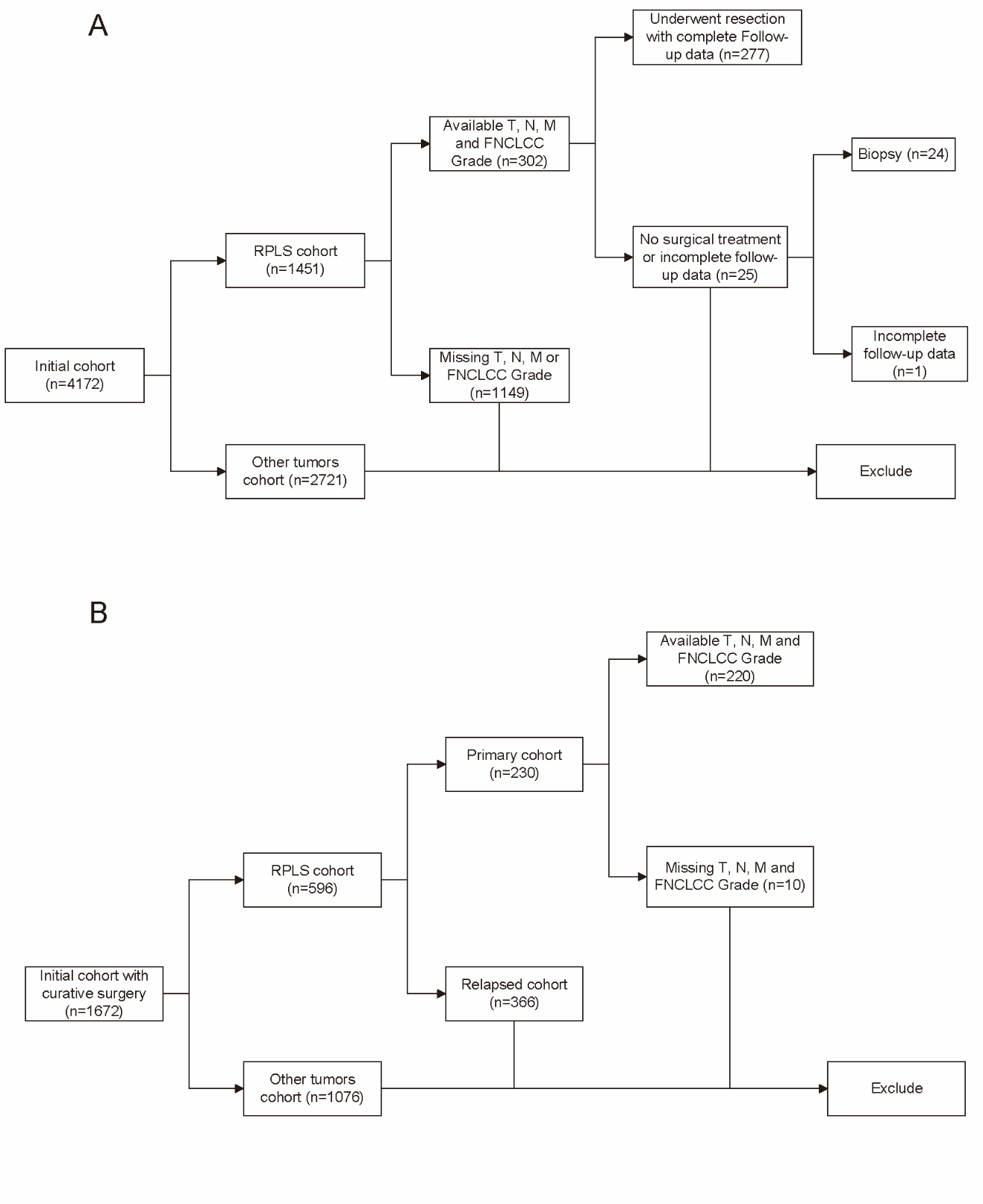


**Supplementary Figure 1.** Flowchart of patient selection in two Cohorts. (A) SEER Cohort, (B) FDZS Cohort.


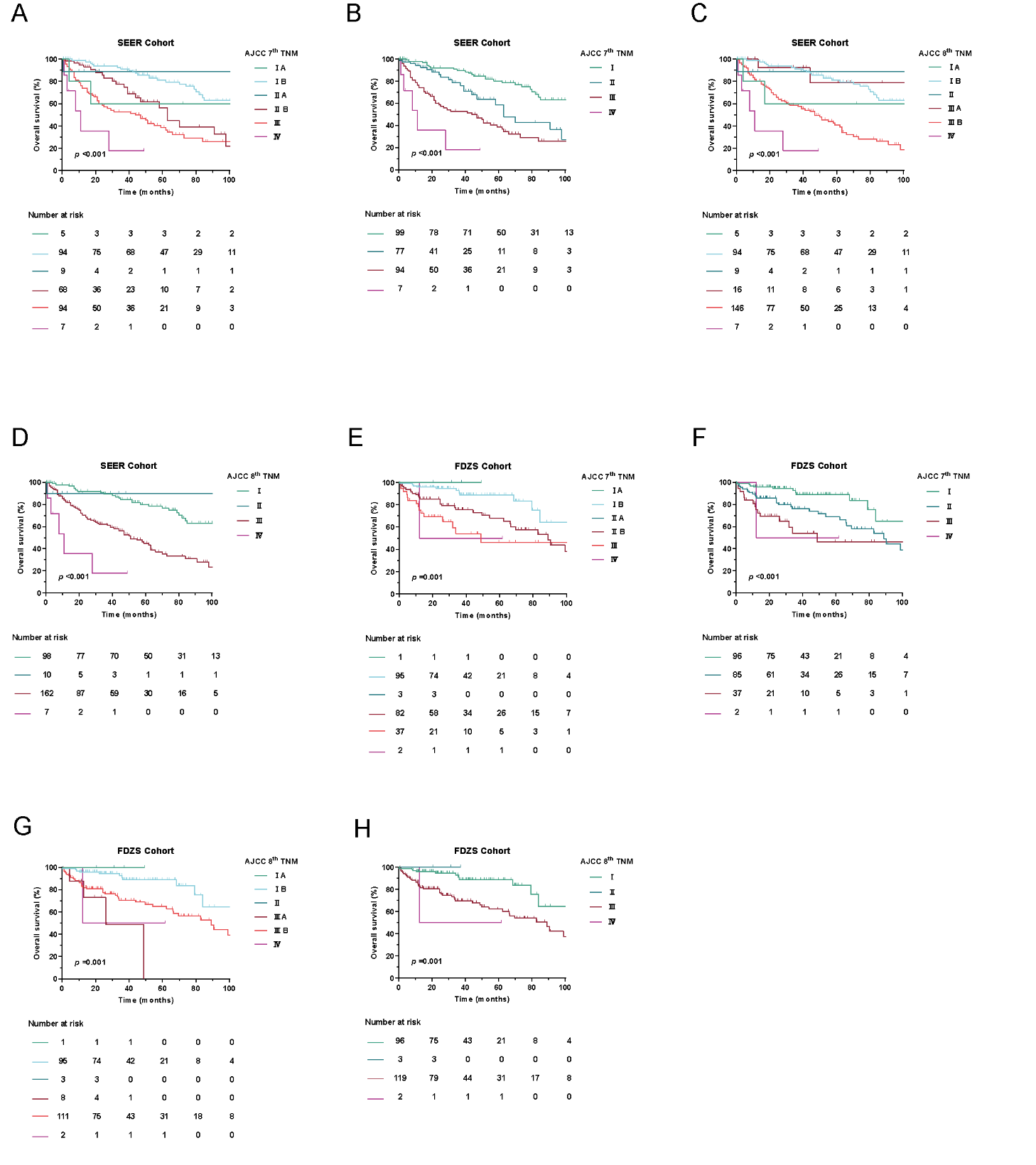


**Supplementary Figure 2.** Kaplan-Meier curves of OS according to AJCC TNM staging in two Cohorts. (A,B) AJCC 7^th^ TNM staging for SEER Cohort, (C,D) AJCC 8^th^ TNM staging for SEER Cohort, (E,F) AJCC 7^th^ TNM staging for FDZS Cohort, (G,H) AJCC 8^th^ TNM staging for FDZS Cohort.


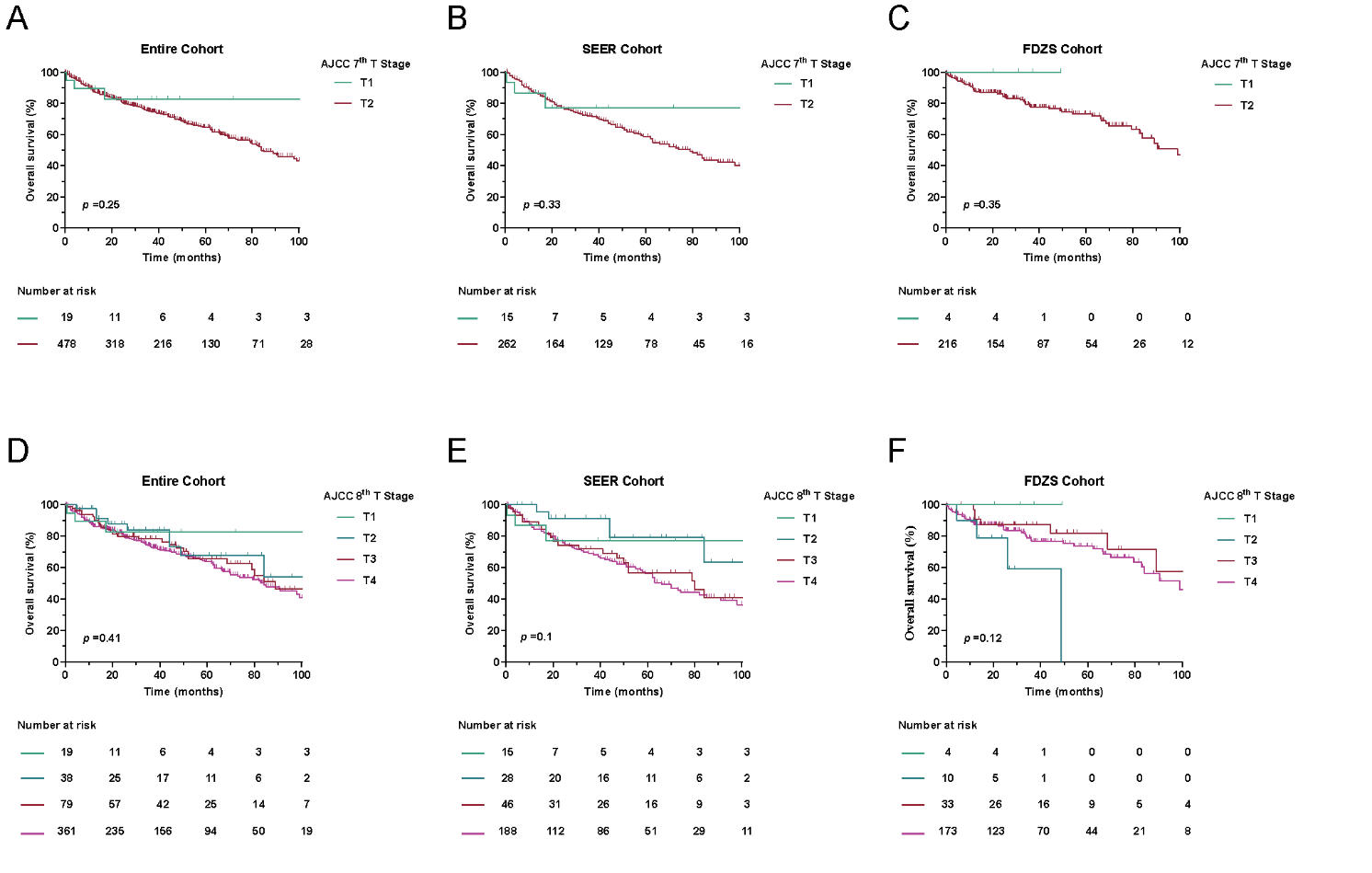


**Supplementary Figure 3.** Kaplan-Meier curves of OS by AJCC 7^th^ T staging. (A,B,C) and AJCC 8^th^ T staging (D,E,F) among different Cohorts. (A,D) Entire Cohort, (B,E) SEER Cohort, (C,F) FDZS Cohort.


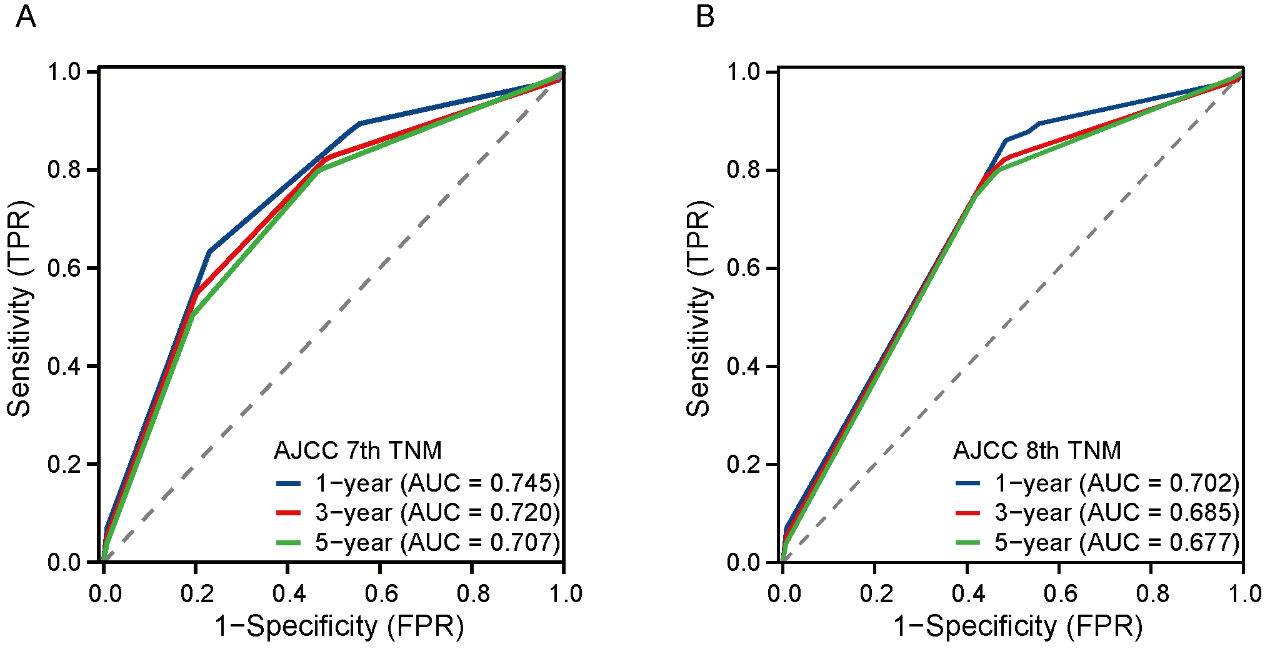


**Supplementary Figure 4.** Receiver operating characteristic (ROC) curves in predicting 1-, 3-, 5-year OS in RPLS after resection by AJCC TNM staging. (A) AJCC 7^th^ TNM staging, (B) AJCC 8^th^ TNM staging.

**
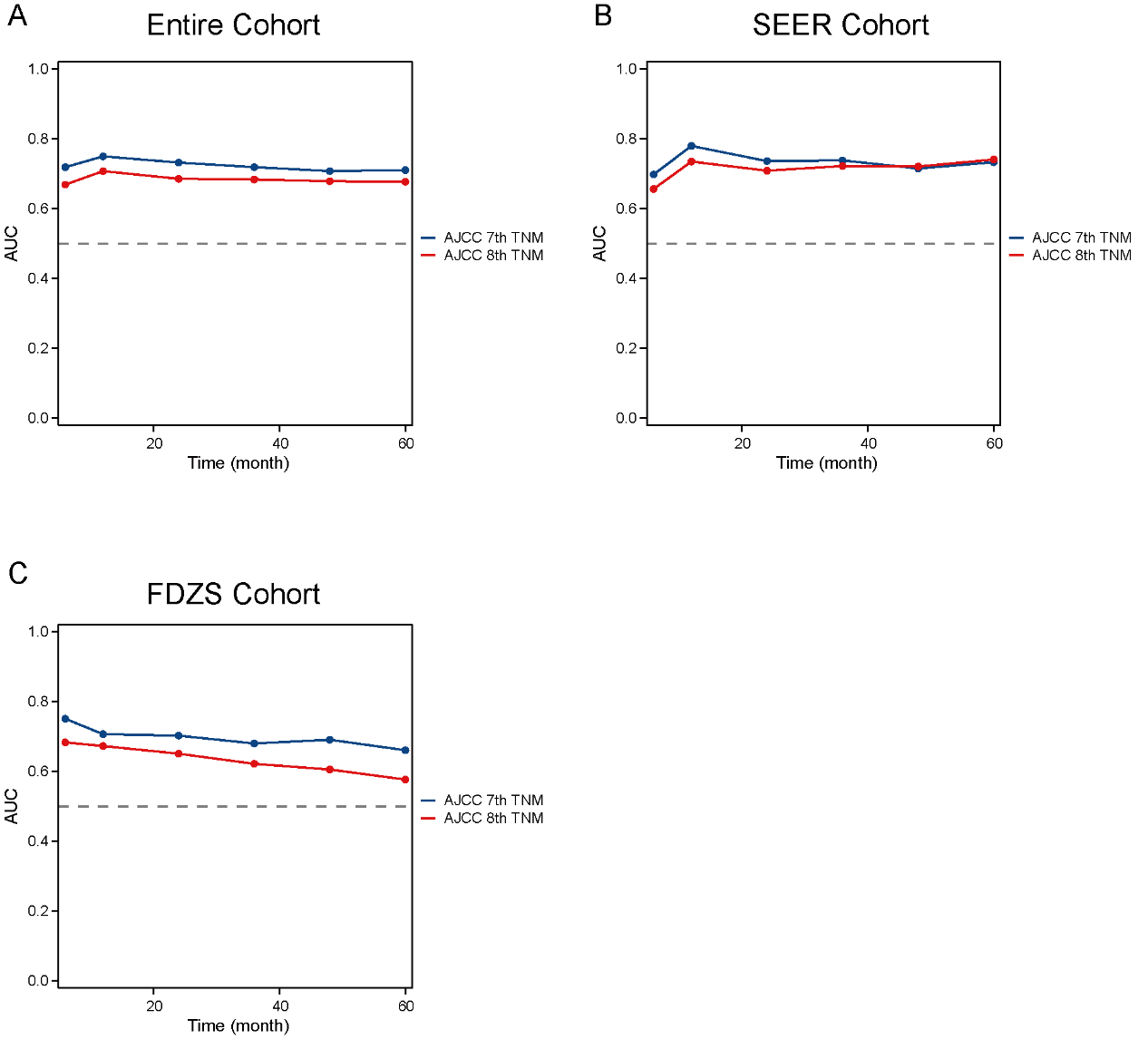
**

**Supplementary Figure 5.** Time-dependent AUC for the 7th and 8th Edition of TNM staging system among three cohorts. (A) Entire Cohort, (B) SEER Cohort, (C) FDZS Cohort.

**
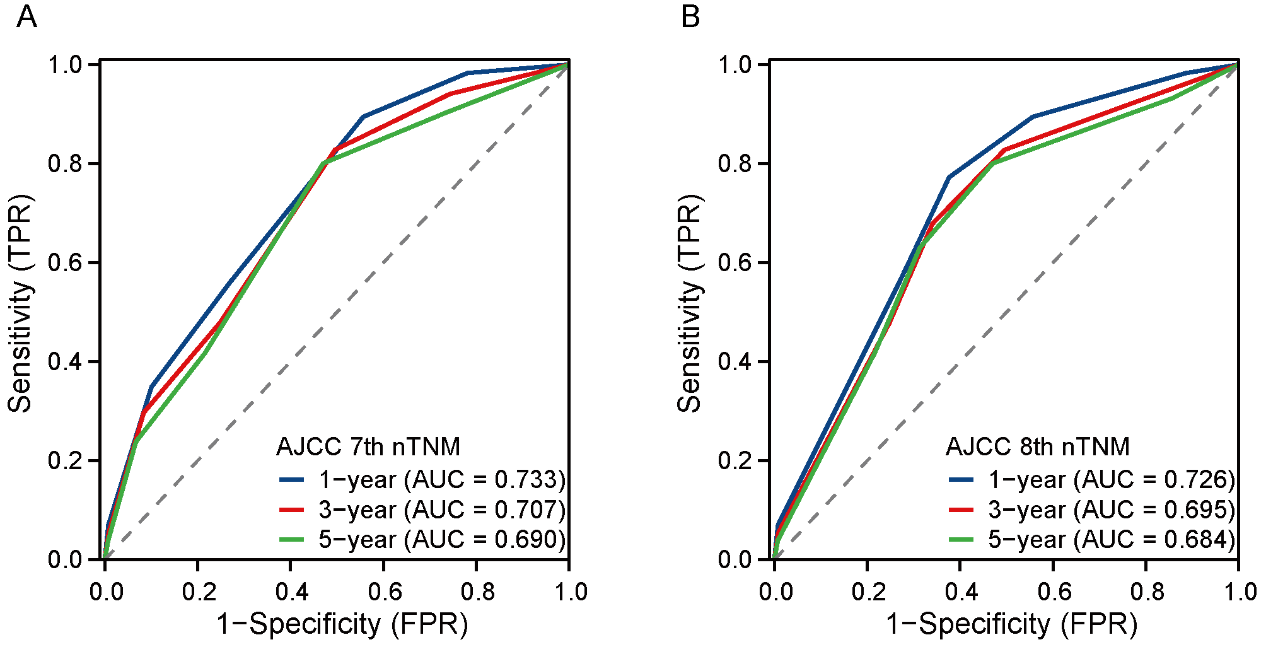
**

**Supplementary Figure 6.** Receiver operating characteristic (ROC) curves in predicting 1-, 3-, 5-year OS in RPLS after resection by modified AJCC TNM staging. (A) AJCC 7^th^ nTNM staging, (B) AJCC 8^th^ nTNM staging.


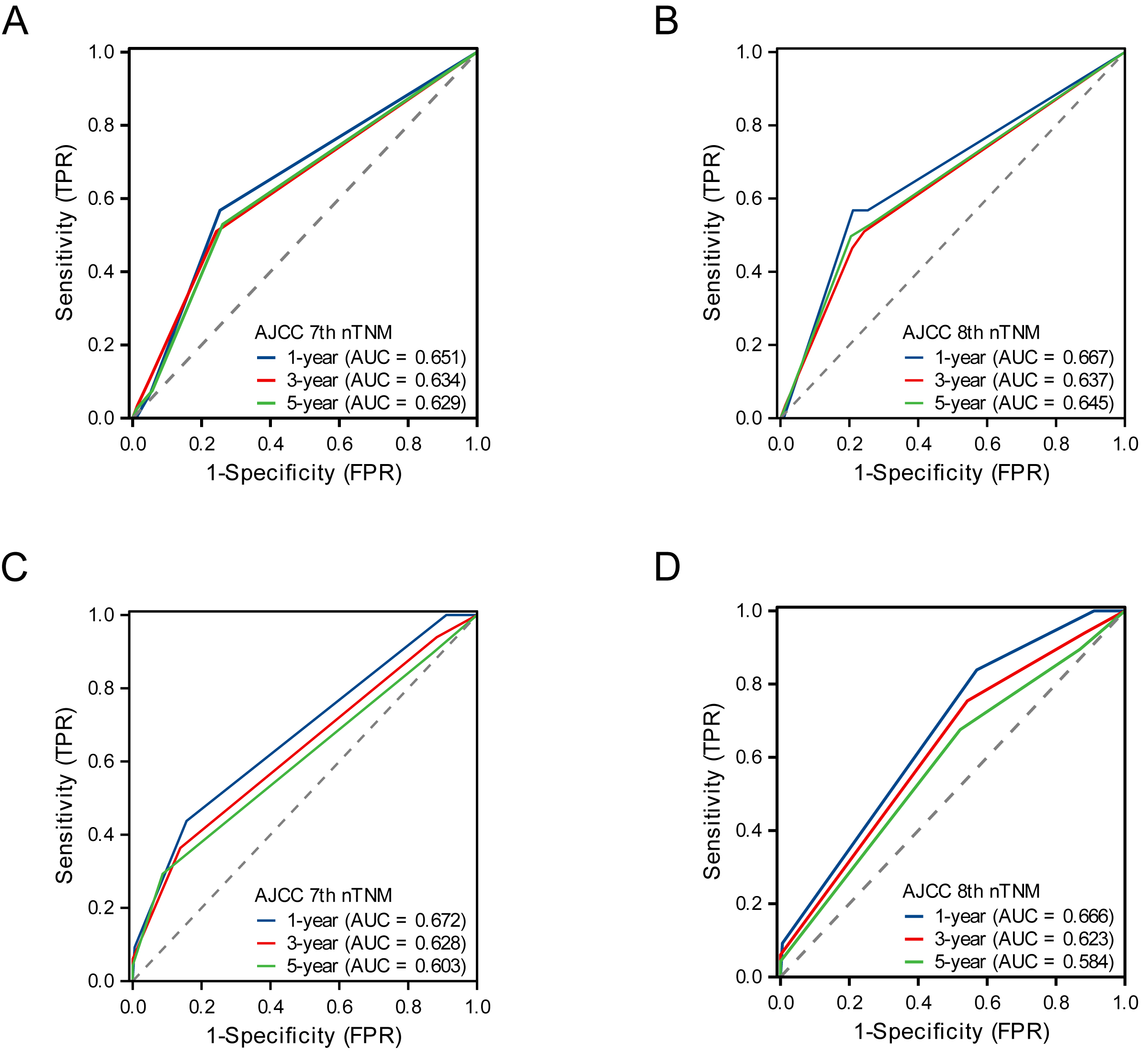


**Supplementary Figure 7.**Receiver operating characteristic (ROC) curves in predicting 1-, 3-, 5-year OS in H1 (WDLPD, Mixed liposarcoma and ‘liposarcoma,NOS’) and H2 (DDLPS, MLPS and PLS) after resection by modified AJCC TNM staging. (A) AJCC 7^th^ nTNM staging for H1, (B) AJCC 8^th^ nTNM staging for H1, (C) AJCC 7^th^ nTNM staging for H2, (D) AJCC 8^th^ nTNM staging for H2.
